# Community Pharmacists' Knowledge, Attitudes, and Practice of Herbal Medicines in Asir Region, Kingdom of Saudi Arabia

**DOI:** 10.1155/2018/1568139

**Published:** 2018-08-30

**Authors:** Abdulrhman Alsayari, Dalia Almghaslah, Arwa Khaled, Sivakumar Annadurai, Mona Ali Alkhairy, Hagar Abdulhadi Alqahtani, Boshra Abduh Alsayed, Rawabi Mohammed Alasiri, Abeer Mohammed Assiri

**Affiliations:** College of Pharmacy, King Khalid University, P.O. Box 1882, Abha 61441, Saudi Arabia

## Abstract

**Background:**

A dramatic increase in the use of natural products and herbal medicines has been observed globally. Simultaneously, there has been an increase in safety concerns regarding the extensive use of these herbal remedies among health care practitioners. The urban and rural populations of Saudi Arabia still rely on traditional Arabic herbal medicines for the treatment of various diseases.

**Objectives:**

This study aimed to evaluate community pharmacists' knowledge, attitudes, and practices of herbal medicines in the Asir region, Saudi Arabia.

**Methods:**

An online cross-sectional study was conducted among 233 community pharmacists using a structured questionnaire.

**Results:**

Pharmacists showed considerable knowledge of the indications of herbal products, with an average score of 84% correct answers, total* P* value < 0.05 and < 0.001. They were also knowledgeable about contraindications, side effects, and interactions, with an average score of 75% correct answers, total* P* value < 0.05 and < 0.001. Community pharmacists had a positive attitude towards herbal products, as 71% of them “agreed” or “strongly agreed” that herbal products were efficacious and 77% of them “agreed” or “strongly agreed” that those products should be sold only in a pharmacy. Herbal products were “often” or "always” dispensed by 67.3% of pharmacists in a pharmacy.

**Conclusion:**

Pharmacists generally exhibited good knowledge, a positive attitude, and effective practice towards herbal products. However, continuing education programs are needed to train pharmacists in providing client counseling on herbal medicine usage and dispensing them.

## 1. Introduction

A dramatic increase has been noted in the global consumption of natural products and herbal medicines in the past three decades [1]. The surge in the use of herbal medicines world-wide has caught the attention of researchers, healthcare practitioners, and regulatory agencies [2]. The belief that herbal medicines promote healthier living is cited as a prime reason for its popularity in developed countries. In developing countries, 80% of the population relies on traditional herbal medicines for their primary healthcare needs [3].

Saudi Arabia, one of the richest oil countries in the Middle East, has more than 30 million people. While the population lives in both urban and rural areas, they still use traditional Arab medicines for health care and treatment of diseases [4]. This is understandable, since Saudi Arabia has one of the richest areas of biodiversity in the Gulf, which consists of an admixture of elements from Asia, Africa, and the Mediterranean regions. The mountainous area of southwestern Saudi Arabia is distinctive for the richness of its vegetation and its species diversity [5]. Approximately 70% of the Saudi flora are found in the area called Al-Sawdah Mountain, close to the city of Abha, the capital of Asir province [6, 7].

Herbal remedies are available to residents in Saudi Arabia from herbal remedy shops or retail outlets. Traditional herbal remedies are usually sold in their crude forms through folk medicine shops. The availability of the new herbal remedies in pharmaceutical dosage forms has made them amenable to being dispensed through community pharmacies. Since community pharmacies come under the purview of the Ministry of Health, these herbal medicines are registered. However, a large proportion of unregistered herbal medicines continue to be dispensed through outlets other than pharmacies [8, 9]. The use of unregistered herbal products and the concomitant use of conventional allopathic medicines resulting in drug-herb interactions is a definite cause of concern for health practitioners and supports the dispensing of herbal products through pharmacies.

The Saudi Food and Drug Authority (SFDA) is the regulatory authority for registration, listing and licensing of herbal products in Saudi Arabia. SFDA provides frequent warning alerts regarding the use of unregistered herbal products. For example, recent alerts have focused on unlicensed natural products being sold for weight loss. The SFDA analysis revealed the presence of sibutramine (a restricted pharmaceutical substance) in Magrim Super Diet being sold by street vendors as a natural product for weight loss.

Several studies have been carried out pertaining to the use of herbal remedies in Saudi Arabia. A study on the use of herbs by diabetic patients concluded that physicians need to encourage patients to disclose their herbal use for effective management of the disease [10]. Another study reported the high usage of herbal medicines in the treatment of obesity [11]. Studies pertaining to the role of community pharmacists have been minimal [8, 12]. The need for community pharmacists to be better informed about herbal products and the need for availability of herbal medicinal information resources have been stressed in these studies. However, the studies were restricted to Riyadh, Saudi Arabia.

The objective of the present study was to assess the knowledge, attitude, and practice of community pharmacists in the Asir region of Saudi Arabia known for its rich flora. This area has an estimated population of 2 million people. A recent community-based cross-sectional study conducted in the region concluded that a significant proportion of the surveyed population favored herbal medicines as a treatment of choice for epilepsy [13]. In light of this finding, our study assumes significance, as it will help us in understanding the challenges faced by the community pharmacists in Asir region.

## 2. Materials and Methods 

### 2.1. Study Design

This study used a prospective cross-sectional self-administered questionnaire, which was carried out in community pharmacies across the Asir region.

### 2.2. Population and Setting

The current study was conducted with community pharmacists practicing in the Asir region, which is in the southern province of Saudi Arabia. The total number of licensed community pharmacies in this region is 438. We intended to recruit one to two pharmacists from each pharmacy.

### 2.3. Sample Size and Sampling Procedure

The sample size was based on the number of community pharmacists in the Asir region (747) and determined by using a Raosoft sample size calculator (http://www.raosoft.com/samplesize.html) with a predetermined margin of error of 5% and a confidence level of 95%. In order to minimize erroneous findings and to increase study reliability, the target sample size was set at 254 pharmacists. Of that 254, 233 responded to the survey, giving a response rate of 91.7%. A nonprobability convenience sampling method was used, including one of the largest chains of community pharmacies in the region with 90 locations, as well as 30 random small pharmacies. The widely spread sites of the chain ensured a geographical distribution of the sample. It is worth mentioning that community pharmacies are usually run by one to two pharmacists or by a pharmacist and an assistant. They work from 8 to 12 hours a day, 6 days a week. The inclusion criteria were as follows: being a licensed pharmacist in Saudi Arabia; working in a community pharmacy in the Asir region, having a minimum of a Bachelor degree certification or higher degree, and being willing to participate in the study. The exclusion criteria were as follows: pharmacy personnel working in other sectors including hospitals, industry, academia, and pharmacy regulation, and community pharmacists who are based outside the Asir region.

### 2.4. Data Collection Form

The structured questionnaire was adapted from previous studies [2, 8, 13]. The questionnaire consisted of 4 domains: demographics/background information, knowledge, attitudes, and practice related to herbal products. The first section of the questionnaire gathered demographic information on the participants, such as age, gender, education level, area of residence, and area of practice/setting. It also asked about the most commonly used herbal medicines. The second section assessed pharmacists'** knowledge** by asking true or false questions regarding the indications, side effects, and contraindications of nine of the most commonly used herbal medicines, such as Ginseng, St John's Wort, and Valerian. The nine herbal medicines were selected based on the findings of previous studies conducted in Saudi Arabia and other studies in the Middle East [2, 8, 14, 15]. The questions were adapted from the studies by Khdour et al. and Alkharfy since these two studies asked questions concerning the safety and efficacy of the nine herbal medicines that are common in the region [2, 8]. The third part of the questionnaire used a five-point Likert scale— (1) strongly disagree; (2) disagree; (3) neutral; (4) agree; and (5) strongly agree— to determine pharmacists'** attitudes **towards herbal medicines. In the last section of the questionnaire, pharmacists were asked to choose a response (never, rarely, sometimes, often, and always) regarding their** practice** of herbal medicines. Attitude and practice statements were adapted from a previously validated data collection tool by Asmelashe Gelayee and colleagues [14]. Assessment of the responses was done blindly. Face and content validity of the final section was discussed and agreed upon by three experts in the field. The questionnaire was piloted with five pharmacists who were representatives of the study population to determine the clarity of the language and the questionnaire structure. The results of the pilot study were not included in the final results. The data collection tool was reviewed and modified based on the feedback received in the pilot. The questionnaire was finalized in the English language in the form of a self-administered web-based questionnaire. The validated questionnaire was delivered to study participants, with the data being collected from October to December 2017.

### 2.5. Statistical Analysis

The collected data were cleared, entered, and analyzed by using the Statistical Package for Social Sciences (SPSS) version 24.0 for Mac. Results were described in terms of frequencies. A Cochran test followed by pairwise was conducted to assess participants' knowledge of the indications, contraindications, and side effects of herbal medicines. The questionnaire was piloted with five pharmacists who were representatives of the study population to determine the clarity of the language and the questionnaire structure. The validated questionnaire was delivered to study participants. The results of the pilot study were not included in the final results.

The Friedman test, followed by pairwise comparisons, was carried out to assess pharmacists' attitudes and practices. P value < 0.05 was considered significant.

### 2.6. Ethical Approval

An ethical clearance was given by the College of Pharmacy, King Khalid University (15/09/2017), and all respondents were asked for their consent before participation in the study. Personal information was reidentified prior to the data analysis.

## 3. Results

A total of 233 pharmacists from various parts of the region participated in the study. Most of the participants (62.2%) were between the ages of 20-29 years [*P *value (0.0001)], with varying degrees of education, including 4.3% with a postgraduate diploma, 91.4% with a Bachelor's degree in pharmacy, and 0.4% with a Master's degree; 1.3% with a Pharm.D.; and none with a Ph.D. [*P* value (0.0001)]. All respondents were male (100%), since females are not allowed to work in community pharmacies. The pharmacists' work experience ranged from 1 to 20 years [*P *value (0.0001)].

The data in**Table 1** suggests that the most common herbal products dispensed through the community pharmacy are Ginseng (79.4%), followed by Ginkgo (44.2%), Garlic (42.1%), Valerian (16.3%), and then other (19.7%) (e.g., Echinacea, devil's claw, horse chestnut, passion flower, and guarana). Additional products sold included Chamomile (9%), Ginger (8.6%), and St John's Wort (4.7%), with the least common herbal products sold being Maca-root (1.3%) and Arnica tincture (1.3%).

### 3.1. Knowledge

Nine herbal drugs— namely, Ginseng, Ginkgo, Valerian, St John's Wort, Ginger, Arnica, Chamomile, Maca-root, and Echinacea— were selected to evaluate the pharmacist's knowledge of herbal products (**Tables 2 and 3**). The pharmacists were asked 11 questions pertaining to the therapeutic use, contraindications, drug-herb interactions, and most common side effects of each of the selected herbal medicines. The pharmacists had significant levels of knowledge of the therapeutic indications, with an average of 84.1% correct answers (*P*< 0.05) (**Table 2**). 70% of the participants could recognize possible side effects, contraindications, and drug-herb interactions as well* (P*< 0.05) (**Table 3**).

### 3.2. Attitude

Pharmacists' beliefs and attitudes toward herbal products were assessed using a five-point Likert scale. In general, pharmacists were found to have a positive attitude towards herbal medicine (**Table 4**,* P* value <0.05 and 0.001). Results found that 60.9% of the respondents “agreed” that herbal medications are efficacious, while the majority (45.1%) of the community pharmacists “strongly agreed” that herbal medicines should only be sold in a pharmacy. Almost half (44.2%) “disagreed” that the use of herbal medicine should be limited to patients who have failed traditional prescription therapy; only 3.4% of the respondents “strongly agreed” with the statement. The majority (79.4%) “strongly agreed” or “agreed” that it is a pharmacist's professional responsibility to provide information about herbal medications, while 47.2% “agreed” that continuing education on herbal medications should be mandatory.

### 3.3. Practice

According to the current practice of dispensing herbal products from Saudi pharmacies, herbal products were reportedly dispensed “often” or “always” by 67.3% of pharmacists (**Table 5**). The majority of respondents (42.1%) “sometimes” use herbal drugs for self-treatment. A total of 85 (36.5%) pharmacists believed that they “always” counsel clients about herbal drug use and 46.8% reported that they “sometimes” get inquiries related to herbal drugs. The total for this section (*P *value <0.05 and 0.001) indicated that pharmacists exhibit good practices towards herbal medicine.

## 4. Discussion

In the past three decades, the global consumption of herbal medicines has noticeably increased and Saudi Arabia is not far behind [1]. Similar to other Middle Eastern countries, the health care system is primarily based on conventional medicine. But traditional remedies in the form of herbal medicines continue to be favored by the population, as evidenced by several studies [10–12]. The present study was conducted in the Asir region, known for its rich flora. Herbal medicine seemed to be the preferred treatment of choice for a significant proportion of the surveyed population, as noted in a recent study conducted in the region [13]. This suggests that the most accessible health care practitioners, pharmacists, are likely to receive more queries pertaining to herbal medicines. Our study will be helpful in understanding the challenges faced by community pharmacists in the region.

We found that the most commonly dispensed herbal products in the region were Ginseng (79.4%), Ginkgo (44.2%), Garlic (42.1%), and Valerian (16.3%). The above-mentioned herbal products have also been cited as the most commonly dispensed herbal products in similar studies [8, 15].

Our data suggests that the community pharmacists were well equipped with knowledge about herbal products, such as their indications (*P *value <0.5), side effects, and contraindications. This finding was similar to other studies [2, 8, 14–16]. However, in contrast to previous studies, our study indicates that the surveyed pharmacists in Asir knew about well-established herb-drug interactions (2/3 of questions were answered correctly). In the absence of data, it is difficult to ascertain the factors that contributed to the pharmacists' significant knowledge. One possible reason could be that they were exposed to a pharmacy curriculum that included herbal medicine courses in the curriculum of the undergraduate pharmacy degrees. For example, the Bachelor in Pharmaceutical Sciences and Pharm D programs offered by King Khalid College of Pharmacy, which is the only college of pharmacy in the Asir region, do contain courses in Pharmacognosy, Photochemistry, Herbal Medicine, Complementary and Alternative Medicine, and Clinical Nutraceuticals. Studies have suggested that need-based education is essential when designing pharmacy curriculum. Hence, countries in which herbal medicine use is common should equip student pharmacists with knowledge of this branch of medicine [17, 18]. Education stakeholders should probably include more evidence-based courses pertaining to this subject. Also, continuing education courses and workshops should be organized to widen pharmacists' knowledge on this topic.

Our study shows that only 36% of the pharmacists “always” provide patient counseling. This could be related to a host of factors, including lack of time due to other duties [12]. Patient counseling should be encouraged to ensure safe use herbal products. Pharmacists should assume responsibility for being lifelong learners and continue to self-reflect and develop in this area of practice.

The study results indicated that 88% of pharmacists “sometimes,” “often,” and “always” dispense herbal products in their pharmacies (*P *value <0.5). A majority of them used herbal products for self-treatment, which was also reported in other studies [2, 8, 14, 16].

Obtaining registered, licensed products from community pharmacies is safer than obtaining them from a “traditional druggist.” The latter have been reported to sell unlicensed ingredients, maintain unsuitable storage conditions, and have limited information about interactions among drugs or diseases, as suggested by the SFDA [19].

Pharmacists in the current study showed positive attitudes towards herbal medicine use, as 71% of them “agreed” or “strongly agreed” that herbal products are efficacious and 77% of them “agreed” or “strongly agreed” that those products should be sold only in a pharmacy. This positive attitude may influence patients to obtain licensed herbal products from registered community pharmacists. The vast majority of pharmacists (73%) in this study were interested in receiving more training and continuing education pertaining to herbal products, similar to other studies [2]. Approximately 80% of them “agreed” or “strongly agreed” that they should be the sole providers of patient counseling on herbal products, considering the fact that community pharmacists are the most accessible healthcare professionals.

## 5. Conclusion

The study was conducted to evaluate community pharmacists' knowledge, attitudes, and practice towards herbal medicine in the Asir region of Saudi Arabia. The pharmacists showed considerable knowledge pertaining to the indications of the herbal products, side effects, contraindications, and drug-herb interactions. Respondents demonstrated positive attitudes towards the use of herbal products and were interested in expanding their knowledge on the topic through continuing education. Additionally, they showed social accountability by assuming responsibility for providing patient counseling on herbal medicines.

This study is not without its limitations. The vast majority of the pharmacists who answered the questionnaire lived in the city (88%) and only a few of them lived in the rural areas where residents predominantly use herbal products. Data collection was limited to a single point of time, so changes over time were not assessed. Furthermore, errors by the pharmacists may have occurred in regard to recollecting some information [2, 8]. All the participants were males, as the regulations in Saudi Arabia do not permit females to work in a community pharmacy.

The use of a self-administered web-based questionnaire might have affected the accuracy of our findings, particularly in the knowledge section, because this method eliminates the opportunity to observe participant behaviour while they complete the survey. As a result, participants could have easily checked the answers to the questions online or asked a colleague at their workplace.

Future research should focus on the use of unlicensed herbal products, the registration of traditional drug stores, and the regulations that control their practice.

## Figures and Tables

**Table 1:** Demographic characteristics of the respondents/background information.

**Characteristics**	**Number (out of total 233)**	**Percentage %**	**Chi square**	***P* value**
**Gender**				
Male	233	100		
Female	0	0		
Age				
20-29	145	62.2	123.1	0.0001*∗∗*
30-39	83	35.6		
40-49	5	2.1		
50-59	0	0		
>60	0	0		
**Education level**				
Post-graduate diploma	10	4.3	1058.5	0.0001*∗∗*
Bachelor degree	219	93.9		
Pharm D	3	1.3		
Master degree	1	0.4		
PhD	0	0		
**Experience**				
<1	4	1.7	296.5	0.0001*∗∗*
1-5	133	57.1		
6-10	74	31.8		
11-15	16	6.9		
16-20	5	2.1		
20	1	0.4		
**Location of the pharmacy**				
City	207	88.8	213.8	0.0001*∗∗*
Village	26	11.2		
**The most commonly dispensed herbal product through the community pharmacy**				
Ginseng	185	79.4	.785	0.376
Ginkgo	103	44.2	.785	0.376
Valerian	38	16.3	132.7	0.000*∗∗*
St John's wort	11	4.7	250.9	0.000*∗∗*
Ginger	20	8.6	207.4	0.000*∗∗*
Arnica	3	1.3	298.6	0.000*∗∗*
Chamomile	21	9	204.2	0.000*∗∗*
Maca|-root	3	1.3	298.6	0.000*∗∗*
Garlic	98	42.1	3.5	0.000*∗∗*
Other (e.g. Echinacea, devil's claw, horse chestnut, passion ower, guarana)	46	19.7	108.4	0.060
**Categories of herbal products that are most commonly dispensed**				
Cough preparations	203	87.1	218.5	0.0001*∗∗*
General Health Tonic (Vitality)	100	42.9	0.333	0.564
Slimming agents	54	23.2	71.05	0.0001*∗∗*
Others (Insomnia, Low Mood, children immunity, Mental alertness)	34	14.6	141.5	0.0001*∗∗*

*∗*p<0.05, *∗∗*p <0.001.

**Table 2:** Pharmacists' knowledge about the indications of selected herbal medicines (n=233).

**Indications**				
**Statement**	**% of correct answer**	**% of incorrect answer**	**Cochrans test**	**Total *P* value**
Echinacea is used to boost immunity	96.1 (224)	3.9 (9)	X2= 206	<0.05*∗* and 0.001*∗∗*
St John's wort is commonly used for mild to moderate depression	96.6 (225)	3.4 (8)		
Arnica is used for minor skin irritation and bruises	85 (198)	15 (35)		
Ginger is used for motion sickness and pregnancy-associated nausea and vomiting	66.5 (155)	33.5 (78)		
Ginkgo is claimed to delay dementia	85.8 (200)	14.2 (33)		
Chamomile is indicated for inflammation, anxiety and insomnia	74.7 (174)	25.3 (59)		

*∗*p<0.05, *∗∗*p <0.001.

**Table 3:** Pharmacists' knowledge about the side effects, contraindications, and drug-herb interactions of selected herbal medicines (n=233).

**Side effects, contraindications, and drug-herb interactions**			**Cochrans test**	**Total *P* value**
**Statement**	**% of correct answer**	**% of incorrect answer**		
Ginkgo can increase the risk of bleeding when combined with warfarin	95.3 (222)	4.7 (11)	X^2^= 307.356	<0.05*∗* and 0.001*∗∗*
St John's wort may increase blood digoxin level	21.5 (50)	78.5 (183)		
Valerian should be used cautiously in patients using benzodiazepines	82.8 (193)	17.2 (40)		
Maca- root should be avoided in patients with goiter	77.3 (180)	22.7 (53)		
Ginseng may increase blood pressure	88.8 (207)	11.2 (26)		

*∗*p<0.05, *∗∗*p <0.001.

**Table 4:** Pharmacists' attitude towards the use of herbal products (n=233).

**Attitudes**						**Friedman test**	**Total *P* value**
**Statement**	**Strongly Disagree n (%)**	**Disagree n (%)**	**Neutral n (%)**	**Agree n (%)**	**Strongly Agree n (%)**		
Herbal medications are efficacious.	7 (3)	4 (1.7)	55 (23.6)	142 (60.9)	25 (10.7)		
Herbal medicines should be sold only in a pharmacy.	16 (6.9%)	10 (4.3)	23 (9.9)	79 (33.9%)	105 (45.1)		
The use of herbal medicine should be limited only to patients who have failed traditional prescription therapy.	26 (11.2)	103 (44.2)	61 (26.2)	35 (15)	8 (3.4)	X^2^= 402.9	<0.05*∗* and 0.001*∗∗*
Providing information about herbal medication is a pharmacist's professional responsibility.	14 (6)	4 (1.7)	30 (12.9)	107 (45.9)	78 (33.5)		
Continuing education on herbal medications should be mandatory.	12 (5.2%)	6 (2.6%)	42 (18%)	110 (47.2%)	63 (27%)		

*∗*p<0.05, *∗∗*p <0.001.

**Table 5:** Pharmacists' practice towards the use of herbal products (n=233).

**Practice**						**Friedman test**	**Total *P* value**
**Question**	**Never n (%)**	**Rarely n (%)**	**Sometimes n (%)**	**Often n (%)**	**Always n (%)**		
1. Do you dispense herbal drugs in your pharmacy?	12 (5.2)	10 (4.3)	53 (22)	71 (30)	87 (37.3)	X^2^=**104.7**	**<0.05** **∗** ** and 0.001** **∗** **∗**
2. Do you use herbal drugs for self-treatment?	5 (2.1)	7 (3)	98 (42.1)	72 (30.9)	51 (21.9)		
3. Do you counsel your clients about using of herbal drugs?	5 (2.1)	7 (3)	74 (31.8)	62 (26.6)	85 (36.5)		
4. Do you get inquiries related to herbal drugs?	12 (5.2)	17 (7.3)	109 (46.8)	58 (24.9)	37 (15.9)		

*∗*p<0.05, *∗∗*p <0.001.

## Data Availability

The data used to support the findings of this study are available from the corresponding author upon request.
